# An Enhanced Approach for Economic Evaluation of Long-Term Benefits of School-Based Health Promotion Programs

**DOI:** 10.3390/nu12041101

**Published:** 2020-04-16

**Authors:** John Paul Ekwaru, Arto Ohinmaa, Paul J. Veugelers

**Affiliations:** School of Public Health, University of Alberta, 3-50 University Terrace, 8303–112 St, Edmonton, AB T6G 2T4, Canada; aohinmaa@ualberta.ca (A.O.); paulus@ualberta.ca (P.J.V.)

**Keywords:** economic evaluation, school health, health promotion, chronic disease prevention, economic evaluation method, cost-effectiveness, return on investment, weight status, physical activity, nutrition, vegetables, fruits

## Abstract

Chronic diseases constitute a tremendous public health burden globally. Poor nutrition, inactive lifestyles, and obesity are established independent risk factors for chronic diseases. Public health decision-makers are in desperate need of effective and cost-effective programs that prevent chronic diseases. To date, most economic evaluations consider the effect of these programs on body weight, without considering their effects on other risk factors (nutrition and physical activity). We propose an economic evaluation approach that considers program effects on multiple risk factors rather than on a single risk factor. For demonstration, we developed an enhanced model that incorporates health promotion program effects on four risk factors (weight status, physical activity, and fruit and vegetable consumption). Relative to this enhanced model, a model that considered only the effect on weight status produced incremental cost-effectiveness ratio (ICER) estimates for quality-adjusted life years that were 1% to 43% higher, and ICER estimates for years with chronic disease prevented that were 1% to 26% higher. The corresponding estimates for return on investment were 1% to 20% lower. To avoid an underestimation of the economic benefits of chronic disease prevention programs, we recommend economic evaluations consider program effects on multiple risk factors.

## 1. Introduction

Chronic diseases are the leading cause of death and disease burden in almost all countries worldwide, except in Africa. It is projected that chronic diseases will account for 7.63 million deaths (66.7% of all deaths) worldwide in 2020. In Canada, chronic diseases account for 89% of all deaths [1] and more than $93 billion in direct and indirect health care costs per year [2]. While there are a number of causes of chronic diseases, at least 80% of all cases of type 2 diabetes and cardiovascular disease (CVD), and 40% of all cases of cancer in Canada can be prevented through the adoption of healthy diets, regular physical activity, and avoidance of tobacco products [3]. Despite the issuing of public health recommendations for diet and active living to reduce the burden of chronic diseases, recent studies show that 74% of Canadians consume less than the recommended number of servings of vegetables and fruit [4], 85% of adults are not meeting recommendations for physical activity [5], and 62% have excess body weight [6]. Among 9–13-year-olds, only 32% of girls and 38% of boys meet recommended intake levels of vegetables and fruit [7], and only 7% of Canadian children meet recommended activity levels [8]. 

Because the foundation for the development of obesity and chronic diseases are laid early in life [9,10,11] and healthy behaviors developed in childhood are commonly maintained throughout the lifetime [12], promotion of healthy eating and active living (HEAL) is considered more effective when targeting children [13] and schools are the primary setting to reach nearly all children. Successful school-based HEAL promotion programs are therefore needed to address childhood obesity and consequent chronic diseases. 

Though there is evidence on school-based programs that have been effective in improving HEAL behaviors and preventing obesity [13], it is essential for policy and decision-makers across the health and education sectors to justify the resources needed for the implementation of school-based health promotion programs. In addition to the effectiveness of these programs in improving HEAL behaviors, preventing obesity and adverse health outcomes, policy-makers and stakeholders have to consider program cost, feasibility, acceptability, and sustainability when making policy decisions. To allow for evidence-based decisions, decision-makers also need to know the short- and long-term costs, cost-effectiveness, and return on investments (ROI) of school-based health promotion programs. So far, the most commonly used models in the evaluation of long-term benefits of health promotion programs such as ACE-obesity [14], WHO CDP model [15], NICE-obesity [16] and other similar models [17,18,19,20], rely on simulating program effects through weight status. However, evaluations based on weight status alone may not capture the potential direct health effects of nutrition and activity levels that are not translated into body weight status, resulting in potential underestimation of the true benefits of these programs. There is, therefore, a need for models that can incorporate both the intervention benefits through weight status and any potential direct effects from targeted behavioral risk factors to chronic disease. Models such as the RIVM-CDM model [21], allow for the evaluation of multiple targeted risk factors but assume independence in the distribution of risk factors. The objectives of this manuscript were to (1) propose an approach that incorporates program effects on multiple risk factors while allowing for interdependencies between the risk factors, (2) apply the proposed approach to develop a model that incorporates program effects on four risk factor (i.e., weight status, physical activity, and fruit and vegetable consumption levels) that are commonly targeted by school-based health promotion programs, and (3) apply the developed model in a simulated hypothetical program. 

## 2. Methods

### 2.1. Proposed Approach

The proposed approach involves developing a model that starts with the short-term effects of a given program on the joint distribution of risk factors observed at a chosen model starting age. Then, using transition probabilities of the joint distribution of the risk factors in the general population to propagate the observed short-term effects in a lifetime, while using the established effects of the risk factors on chronic diseases to incorporate the probabilities of developing chronic diseases. Lastly, use the established effects of the risk factors and chronic diseases on mortality to incorporate the probability of death.

### 2.2. A Model to Incorporate Program Effects on Weight Status, Physical Activity, and Fruit and Vegetables Consumption Levels 

In this paper, we specifically developed a model that incorporates health promotion program effects on four risk factors (i.e., weight status, physical activity, and fruit and vegetable consumption levels). We chose these four risk factors because they are the targets of most school-based health promotion programs, in addition to the availability of longitudinal data to estimate their joint transition probabilities.

The model includes 793 Markov states based on; 3 weight status categories (normal weight, overweight, and obesity), 2 categories each (meeting and not meeting recommendations) for physical activity, fruit and vegetable consumption, and 33 chronic disease status (no chronic disease and 32 chronic diseases), plus an absorbing state (died). Only 32 chronic diseases for which the Global Burden of Disease Study (GBD) reported evidence on the relationship to the four risk factors were considered [22] (Table 1). Relative risk estimates linking the 32 chronic diseases to the four risk factors were obtained from the supplementary tables of the GBD Lancet publication [22]. We only included relationships for which the relative risks were statistically significant (Table 1). 

### 2.3. Model Inputs

#### 2.3.1. Program Effects

The joint probability, $\vartheta_{wpfv}^{\left(*\right)}$, of being in a given combined classification of the four risk factor levels at age 10 years, given a particular program’s effects on weight status ($\alpha_{w}$), physical activity level ($\psi_{p}$), fruit ($\lambda_{f})$ and vegetable ($\tau_{v}$) consumptions levels were incorporated in the model using the following composite function for any given number of risk factors, R.

(1) $$\vartheta_{k_{1}\ldots k_{R}}^{\left(*\right)}=\left(f_{R}\circ f_{R-1}\ldots f_{2}\circ f_{1}\right)\left(\vartheta_{k_{1}\ldots k_{R}}^{\left(0\right)}\right)$$

(2) $$f_{r}\left(\vartheta_{k_{1}\ldots k_{R}}\right)=\frac{\xi_{k_{r}}\vartheta_{k_{1}\ldots k_{R}}\sum_{k_{r}}\vartheta_{k_{1}\ldots k_{R}}}{\sum_{k_{r}}\xi_{k_{r}}\vartheta_{k_{1}\ldots k_{R}}}\;,\;\forall_{r\epsilon \left\{1,2,\ldots ,R\right\}}$$

where

$\xi_{k_{r}}$ = Effect of the program on the odds of being in the $k^{th}$ status of risk factor r

$\vartheta_{k_{1}\ldots k_{R}}^{\left(0\right)}$ = is the joint probability of being in a given combination of status levels ($k_{1}\ldots k_{R}$) of the R risk factors under no program effect (base case)

The following notations are used through the manuscript for the specific simulation example.

$\alpha_{w}$ = Effect of the program on weight status, wϵ{0,1,2} for normal weight, overweight and obese, respectively

$\psi_{p}$ = Effect of the program on physical activity level, pϵ{0,1} for not meeting and meeting physical activity levels, respectively

$\lambda_{f}$ = Effect of the program on fruit consumption, fϵ{0,1} for not meeting and meeting fruit consumption levels, respectively

$\lambda_{v}$ = Effect of the program on vegetables consumption, vϵ{0,1} for not meeting and meeting vegetable consumption levels, respectively

$\vartheta_{wpfv}^{\left(0\right)}$—The joint probability of being in a given combined classification of weight status (w), physical activity level (p), fruit (f) and vegetable (v) consumption levels at age 10 years, under no program effect (base case).

Table S1 presents the joint distribution of weight status, physical activity level, fruit and vegetable consumptions levels among under 14-year old’s in Canada that we used as the initial joint probability, $\vartheta_{wpfv}^{\left(0\right)}$, at age 10 years, under no program effect. These estimates were obtained from the analysis of the Canadian Community Health Survey (CCHS) data for 2007 to 2014 annual cycles [23]. 

#### 2.3.2. Joint Transition Probabilities of Weight Status, Physical Activity, and Fruit and Vegetable Consumption Levels 

Estimates of the joint transition probabilities of the four risk factors were obtained from an analysis of longitudinal data from a Canadian cohort called the National Population Health Survey (NPHS), that follows participants 12 years of age or older and collected data every two years [24]. The NPHS target population includes household residents in all provinces and territories, except persons living on Indian Reserves, on Canadian Forces Bases, and in some remote areas [24].

A joint multivariate conditional logit model, using an approach proposed by Westfall et al. [25], was fitted to the NPHS data to estimate the joint transition probabilities of the four risk factors. In this joint model, the independent variables/covariates were; sex, age, weight status, physical activity, fruit and vegetable consumption levels at a given time point, and the joint outcomes were; weight status, physical activity, and fruit and vegetable consumption levels in the next assessment (two years later). The final parsimonious model was obtained using a stepwise model selection process, considering up to four-way interactions for both outcome and explanatory variables. Parameter estimates of the fitted joint model (Table S2) were then used in the Markov model to obtain sex and age-specific joint transition probabilities of the four risk factors at each stage. Reference values used to define meeting physical activity, fruit and vegetable consumption levels are presented in Table S7.

#### 2.3.3. Conditional Probability of Dying Given Weight Status, Physical Activity Level, Fruit Consumption, Vegetable Consumption, and Chronic Disease Status

These probabilities were estimated based on the general Canadian population mortality hazards by sex and age, extracted from the Canadian life table [26] and the effects of chronic diseases, weight status, physical activity level, and fruit and vegetable consumption levels on all-cause mortality [27,28,29,30,31,32,33,34,35,36,37,38,39,40] (Tables S3 and S4). Because the published effects of chronic diseases and the four risk factors on all-cause mortality were not estimated relative to the general population, the prevalence of the various chronic diseases and distribution of the four risk factors were used to convert the reported mortality rate ratios into standardized mortality ratios (SMR).

For example, at any given age and sex, the SMR (mortality ratio relative to the general population) for weight status was estimated as,

(3) $$SMR_{w}=\frac{MR_{w}}{\sum_{w}P_{w}MR_{w}}$$

where

$MR_{w}$—Mortality rate ratio for weight status w compared to normal weight.

$P_{w}$—Proportion of individuals who are in weight status w.

This estimation approach was used for all rate ratios/relative risks for which the reference category was not the general population.

Assuming no synergistic or antagonistic effects of the four risk factors and chronic diseases on mortality, the mortality hazard at any given combination of age, sex, weight status, physical activity, fruit consumption, vegetable consumption, and chronic disease status was estimated using the following expression.

(4) $$h^{*}=\underset{k\epsilon \left\{w,p,f,v,d\right\}}{\prod }\frac{SMR_{k}}{\sum_{k}P_{k}SMR_{k}}\times H$$

where

$h^{*}$—Conditional mortality hazard given sex, age, weight status (w), physical activity level (p), fruit (f) and vegetables (v) consumption levels and chronic disease status (d). 

$SMR_{k}$—Standardized mortality rate ratio for the $k^{th}$ status of the respective risk factor/disease.

$P_{k}$—Proportion of individuals who are in the $k^{th}$ status of a given risk factor/disease.

H—Age and sex-specific mortality hazard, obtained from the Canadian life table.

wϵ{0,1,2}—For normal weight, overweight and obese, respectively.

pϵ{0,1}—For not meeting and meeting physical activity recommendations, respectively.

fϵ{0,1}—For not meeting and meeting fruit consumption recommendations, respectively.

vϵ{0,1}—For not meeting and meeting vegetable consumption recommendations, respectively.

dϵ{1,2,..32}—For the 32 chronic diseases, respectively. 

Probabilities of developing chronic diseases. 

The probabilities of developing chronic diseases were estimated using published data on incidence rates of chronic diseases in Canada [41], the distributions of weight status, physical activity, and fruit and vegetable consumption in Canada and their established effects (relative risks) on chronic diseases [22]. Incidence rates estimates of the 32 chronic diseases were obtained from the Global Burden of Disease database using their online results tool [41]. Incidence rates for cataract and hypertensive heart disease that were not available in the GBD database were estimated from available age-specific prevalence and mortality rate values using a nonlinear equation proposed by Podgor and Leske [42]. This equation which is nonlinear in incidence was solved using the Newton-Raphson method [43].

For each combination of the four risk factors, we estimated the overall probability of developing a given chronic disease assuming no synergistic or antagonistic effects, i.e., combined effect being a product in the multiplicative scale (relative risks). 

At each stage, the probability of developing a given chronic disease given, weight status (w), physical activity level (p), fruit (f) and vegetable (v) consumptions levels was estimated as

(5) $$p=1-e^{I_{wpfv}^{*}\times t}$$

where,

(6) $$I_{wpfv}^{*}=\underset{k\epsilon \left\{w,p,f,v\right\}}{\prod }\frac{RR_{k}}{\sum_{k}P_{k}RR_{k}}\times I$$

I—Published age and sex-specific incidence of a given chronic disease.

$I_{wpfv}^{*}$—Estimated age and sex-specific incidence of a given chronic disease for given weight status, physical activity level, fruit and vegetable consumption.

t—Markov cycle length in years.

$RR_{k}$—Relative risk of developing a given chronic disease for the $k^{th}$ status of each respective risk factor.

$P_{k}$—Proportion of individuals who are in the $k^{th}$ status of a given risk factor.

### 2.4. Outcomes

We estimated three outcome measures from the model i.e., person-years with chronic diseases, quality-adjusted life years (QALYs) lived, and chronic disease health care costs. For every year lived with excess weight or chronic disease, we assigned a decrement in health utility scores that are based on population preferences for health states on the scale 0 (death) and 1 (full health) producing QALYs lived in a given status. The estimated decrements in health utility scores were obtained from previously published estimates by Schultz et al. [44] and Jia et al. [45] (Table S5). Though the decrements estimated by Jia et al. were based on participants who were 18 years of age or older, we applied the same decrements for all over the age of 10 years. For each given combination of weight status and chronic disease Markov state, the higher of the two QALY decrement estimates was used (weight status or chronic disease).

### 2.5. Health Care Costs Attributable to Chronic Disease 

A prevalence approach was used to estimate per-capita annual direct health care costs attributable to major chronic disease diagnosis categories. The total direct health care costs in Canada in 2016 were extracted from the available open data on National Health Expenditure Trends provided by the Canadian Institute for Health Information (CIHI) [46]. These costs were then proportionally allocated to the diagnosis categories based on the proportions obtained from the Economic Burden of Illness in Canada (EBIC 2008) online tool [47]. Estimates of the prevalence of chronic diseases [41] and the total population of Canada in 2016 [48] were used to estimate the number of prevalent cases and subsequently the attributable annual costs per case (Table S6).

### 2.6. Discounting 

All future costs and health outcomes (up to 84 years) were discounted to their present values using an annual discount rate of 1.5% [49].

### 2.7. Simulated Hypothetical Program

To demonstrate the extent to which an evaluation based on only weight status would underestimate the economic benefits of a given program, we simulated a hypothetical program that is implemented for two years at an average annual cost of $50 per student and results in a 30% reduction in the odds of obesity (i.e., odds ratio = 0.7) in combination with various effects on physical activity, fruit and vegetable consumption. From this simulation, we generated estimates of lifetime incremental effects on QALYs, years with chronic disease, and return on investment, that is the ratio between the program benefits (savings) and program costs, for various combinations of program effects on weight status, physical activity, and fruit and vegetable consumption. The model only incorporates the program effects in changing the joint risk factor distributions the model starting point (age 10), thus assuming no additional effect on transition probabilities.

### 2.8. Sensitivity Analysis

We also performed probabilistic sensitivity analysis (PSA) to incorporate uncertainties in all model parameters simultaneously [50]. In PSA, the prevalence and incidence inputs were assumed to follow a beta distribution, relative risks and mortality rate ratios were assumed to follow log-normal distributions, and parameters of the multinomial model for risk transitions were assumed to follow a normal distribution. We carried out a total of 10,000 simulations, where model parameters were assigned random values drawn from their respective distributions. The 2.5th and 97.5th percentiles from these simulations were used to estimate the 95% confidence intervals.

### 2.9. Statistical Analysis

All statistical analyses of data from the Canadian Community Health Survey (CCHS) and the National Population Health Survey (NPHS) were carried out using SAS 9.4 [51]. The economic model was implemented in TreeAge Pro 2020, (Williamstown, MA, USA) [52].

## 3. Results

Parameter estimates of the fitted joint multivariate model of the four risk factors are presented in Table S2. The final parsimonious model included two-way and three-way interactions between the dependent variables, indicating interdependencies between weight status, physical activity, fruit consumption, and vegetable consumption. 

To demonstrate the extent to which an evaluation based on only weight status would underestimate the economic benefits of a given program, we generated estimates of lifetime incremental effects on QALYs, years with chronic disease, and return on investment for various combinations of program effects on weight status, physical activity, and fruit and vegetable consumption. Table 2, Table 3 and Table 4 present partial results for a hypothetical program that is implemented for two years at an average annual cost of $50 per student and results in a 30% reduction in the odds of obesity (i.e., odds ratio = 0.7) in combination with various effects on physical activity, fruit and vegetable consumption. Table 2 shows that an evaluation that considers only a 30% reduction in the odds of obesity produces higher values of the incremental cost-effectiveness ratio (ICER) for QALYs gained relative to evaluations that also consider the effects on physical activity and fruit and vegetable consumption. Estimated ICERs based on obesity reduction alone ranged from 1% higher, if the program had a modest 1% increase in the odds of meeting recommendations for only vegetables, to 43% higher if the program doubled the odds of meeting recommendations for all the three behavior factors in addition to reducing the odds of obesity (Table 2). 

Similarly, the ICER for years with chronic disease prevented would range from 1% higher if the program had a modest 1% increase in the odds of meeting the recommended vegetable consumption, to 26% higher if it doubled the odds of meeting all three behavioral risk factors in addition to reducing the odds of obesity (Table 3).

Underestimation of the return on investment ranges from 1% if the program had a modest 1% increase in the odds of meeting recommendations only for vegetable consumption to 20% if it doubled the odds of meeting recommendations for all three behavior factors in addition to reducing the odds of obesity (Table 4). 

## 4. Discussion

We developed a comprehensive model to estimate the economic benefits of school-based health promotion that considers program effects on multiple targeted risk factors such as nutrition and physical activity in addition to the effects on body weight status. In our demonstration example, we show that such a comprehensive model provides substantially more favorable estimates of cost-effectiveness and return on investment than the commonly applied models [14,15,16,17,18,19,20] that only consider the program effects on only one risk factor like weight status. In other words, these commonly applied models result in underestimation of the true economic benefits. This is because these models essentially assume that all the targeted modifiable risk factors (nutrition and physical activity) affect chronic diseases only through their effects on body weight. Herewith they ignore the independent effects of nutrition and physical activity on chronic disease, over and above the effects of body weight, that have been shown to exist in meta-analyses of studies that adjusted for body mass index [53,54]. For this demonstration, we calculated ICERs using only the program costs, but the observed underestimation also applies to ICERs calculated after future health care cost savings are deducted from program costs.

The estimates presented in Table 2, Table 3 and Table 4, demonstrate that economic evaluations based on program effects on body weight alone, as commonly done [14,15,16,17,18,19,20], result in underestimation of long-term program benefits. We, therefore, recommend that future economic evaluations consider program effects on multiple outcomes and that those evaluations that considered a program effect on only a single outcome be considered an underestimation. 

With 89% of all deaths being attributable to chronic diseases [1] and with more than $93 billion in annual direct and indirect health care costs (2), chronic diseases constitute a tremendous public health burden to Canada, as they are globally [3]. Public health decision-makers are therefore in desperate need of intervention programs that are effective and cost-effective. The present paper is essential to them in that it illustrates that many of the studies to date, have underestimated the true cost-effectiveness of the programs evaluated. One could even make the point that the comprehensive estimates of the economic benefits described in the present paper still represent underestimates because there may be more nutritional and other activity risk factors involved: In addition to vegetables and fruit, also whole grains, nuts and seeds, milk, processed meat, red meat, and sugar-sweetened beverages have been shown to be health care costs drivers, totaling $13.8 billion per year for Canada [55]. In addition to lack of physical activity, sedentary activity and inadequate sleep have also been shown to contribute to health care costs [56,57]. The unfortunate reality is that a full spectrum of nutritional and movement information is rarely collected. Instead, the information is typically limited to vegetables and fruit consumption, physical activity and body weight, which we had used in the present study. However, the models used in the present study can be extended to include those other nutritional and movement risk factors, as well as other behavioral risk factors that programs may target such as smoking and alcohol use.

In addition to the notion that estimates of economic benefits to date represent underestimates, the present study is also essential to public health decision-makers in that it shows that multi-component or comprehensive interventions are more likely to lead to economic benefits. As each component of nutrition, physical activity, and body weight have independent contributions for cost-effectiveness and return on investment, program designers and implementers are encouraged to target each of the three, rather than, for example, only nutrition or only physical activity. This applies as much to school programs as it does to community-based, occupational and clinical programs. 

A strength of the present study is that we took a prospective incidence modeling approach and used the most reliable available input estimates. Incidence, prevalence and relative risk estimates were all obtained from the Global Burden of Disease Study, which provides a comprehensive assessment of risk factor exposure and attributable burden of disease using an extensive body of published literature and several updated data sources [22]. The joint transition probabilities were based on a comprehensive longitudinal survey that has been collecting data in all Canadian provinces and territories every two years since 1994 [24]. However, what applies to all economic models also applies to our models in that they represent a simplification of reality and rely on a number of assumptions and model inputs obtained from various sources [58]. We applied health utility score decrements due to weight status that were estimated for the 18+ years population to the 10–17 years age group. This may have underestimated the QALYs if the decrements are smaller for those under 18 years old. However, we do not expect this to change findings on the relative comparisons as the underestimation applies equally to the compared scenarios. As a limitation, we should also note that the transition probabilities were calculated based on self-reported rather than measured nutrition, physical activity and body weight which is prone to error. We are not aware of any better longitudinal data sets to derive the transition probabilities from. It is for this reason that we make our parameter estimates available (Table S2) for other researchers who may wish to use them. Though we used a cohort-based Markov model in our demonstration, the model can also be implemented using microsimulation which has more flexibility in incorporating aspects such as co-morbidities without increasing the model states substantially.

## 5. Conclusions 

Economic evaluation of long-term benefits of health promotion programs, particularly comprehensive programs that target multiple risk factors, should be comprehensive and not based on only their effects on weight status. Current estimates of cost-effectiveness and return of investment, that may have been already considered in policy decisions, likely represent significant underestimations of the true economic benefits.

## Figures and Tables

**Table 1:** Chronic diseases and linked risk factors (X).

	Risk Factor			
Chronic Disease	Weight Status	Physical Activity	Fruit Consumption	Vegetable Consumption
Diabetes	X	X	X	
Hypertensive heart disease	X			
Asthma	X			
Ischemic heart disease	X	X	X	X
Ischemic stroke	X	X	X	X
Hemorrhagic stroke	X		X	X
Chronic kidney disease	X			
Leukemia	X			
Osteoarthritis	X			
Gout	X			
Low back pain	X			
Cataract	X			
Gallbladder and biliary diseases	X			
Atrial fibrillation and flutter	X			
Alzheimer’s disease and other dementias	X			
Breast cancer	X	X		
Colon and rectum cancer	X	X		
Esophageal cancer	X		X	
Gallbladder and biliary tract cancer	X			
Kidney cancer	X			
Larynx cancer			X	
Lip and oral cavity cancer			X	
Liver cancer	X			
Multiple myeloma	X			
Nasopharynx cancer			X	
Other pharynx cancer			X	
Non-Hodgkin’s lymphoma	X			
Ovarian cancer	X			
Pancreatic cancer	X			
Thyroid cancer	X			
Tracheal bronchus and lung cancer			X	
Uterine cancer	X			

**Table 2:** Estimated incremental cost-effectiveness ratio (ICER) for Quality Adjusted Life Years (QALYs) gained, considering effects on all four risk factors compared to the effect on weight status alone.

Program Effect on						Considering only Weight Status			Considering All 4 Risk Factors			Ratio (ICER_1_/ICER_2_)	
Overweight (α_1_)	Obesity (α_2_)	Physical Activity (ψ_1_)	Fruit (λ_1_)	Vegetables (τ_1_)	Cost	Incremental Effect	ICER_1_	95% CI	Incremental Effect	ICER_2_	95% CI	Ratio	95% CI
1.0	0.7	1.0	1.0	1.0	99.26	0.0066	15,040	14,174–15,919	0.0066	15,040	14,174–15,919	1.000	1.000–1.000
1.0	0.7	1.0	1.0	1.1	99.26	0.0066	15,040	14,174–15,919	0.0067	14,915	14,054–15,785	1.008	1.007–1.010
1.0	0.7	1.0	1.1	1.0	99.26	0.0066	15,040	14,174–15,919	0.0067	14,860	14,001–15,726	1.012	1.010–1.015
1.0	0.7	1.0	1.1	1.1	99.26	0.0066	15,040	14,174–15,919	0.0067	14,738	13,882–15,600	1.021	1.017–1.025
1.0	0.7	1.1	1.0	1.0	99.26	0.0066	15,040	14,174–15,919	0.0069	14,483	13,655–15,320	1.038	1.037–1.040
1.0	0.7	1.1	1.0	1.1	99.26	0.0066	15,040	14,174–15,919	0.0069	14,367	13,543–15,192	1.047	1.045–1.050
1.0	0.7	1.1	1.1	1.0	99.26	0.0066	15,040	14,174–15,919	0.0069	14,316	13,492–15,141	1.051	1.048–1.055
1.0	0.7	1.1	1.1	1.1	99.26	0.0066	15,040	14,174–15,919	0.0070	14,202	13,386–15,022	1.059	1.055–1.065
1.0	0.7	1.9	1.9	1.9	99.26	0.0066	15,040	14,174–15,919	0.0092	10,768	10,115–11,400	1.397	1.368–1.434
1.0	0.7	1.9	1.9	2.0	99.26	0.0066	15,040	14,174–15,919	0.0093	10,719	10,066–11,350	1.403	1.374–1.441
1.0	0.7	1.9	2.0	1.9	99.26	0.0066	15,040	14,174–15,919	0.0093	10,716	10,059–11,348	1.403	1.374–1.442
1.0	0.7	1.9	2.0	2.0	99.26	0.0066	15,040	14,174–15,919	0.0093	10,668	10,012–11,298	1.410	1.380–1.450
1.0	0.7	2.0	1.9	1.9	99.26	0.0066	15,040	14,174–15,919	0.0093	10,632	9988–11,255	1.415	1.386–1.453
1.0	0.7	2.0	1.9	2.0	99.26	0.0066	15,040	14,174–15,919	0.0094	10,585	9942–11,206	1.421	1.391–1.460
1.0	0.7	2.0	2.0	1.9	99.26	0.0066	15,040	14,174–15,919	0.0094	10,582	9935–11,204	1.421	1.391–1.461
1.0	0.7	2.0	2.0	2.0	99.26	0.0066	15,040	14,174-15,919	0.0094	10,535	9888–11,156	1.428	1.397–1.468

Note: All estimates are based on a 1.5% discounting rate. $\alpha_{1}$—Odds ratio for overweight. $\alpha_{2}$—Odds ratio for obesity. $\psi_{1}$—Odds ratio for meeting the recommended physical activity level. $\lambda_{1}$—Odds ratio for meeting the recommended fruit consumption. $\tau_{1}$—Odds ratio for meeting the recommended vegetable consumption. CI—Confidence interval.

**Table 3:** Estimated incremental cost-effectiveness ratio (ICER) for years with chronic disease prevented, considering effects on all four risk factors compared to the effect on weight status alone.

Program Effect on						Considering only Weight Status			Considering all 4 Risk Factors			Ratio (ICER_1_/ICER_2_)	
Overweight (α_1_)	Obesity (α_2_)	Physical Activity (ψ_1_)	Fruit (λ_1_)	Vegetables (τ_1_)	Cost	Incremental Effect	ICER_1_	95% CI	Incremental Effect	ICER_2_	95% CI	Ratio	95% CI
1.0	0.7	1.0	1.0	1.0	99.26	−0.02642	3757	3169–4328	−0.02642	3757	3169–4328	1.000	1.000–1.000
1.0	0.7	1.0	1.0	1.1	99.26	−0.02642	3757	3169–4328	−0.02655	3738	3154–4303	1.005	1.004–1.007
1.0	0.7	1.0	1.1	1.0	99.26	−0.02642	3757	3169–4328	−0.02659	3733	3152–4296	1.006	1.005–1.009
1.0	0.7	1.0	1.1	1.1	99.26	−0.02642	3757	3169–4328	−0.02672	3714	3138–4275	1.012	1.009–1.015
1.0	0.7	1.1	1.0	1.0	99.26	−0.02642	3757	3169–4328	−0.02705	3670	3101–4223	1.024	1.021–1.026
1.0	0.7	1.1	1.0	1.1	99.26	−0.02642	3757	3169–4328	−0.02718	3652	3087–4203	1.029	1.025–1.033
1.0	0.7	1.1	1.1	1.0	99.26	−0.02642	3757	3169–4328	−0.02721	3647	3085–4195	1.030	1.026–1.034
1.0	0.7	1.1	1.1	1.1	99.26	−0.02642	3757	3169–4328	−0.02735	3629	3072–4175	1.035	1.030–1.041
1.0	0.7	1.9	1.9	1.9	99.26	−0.02642	3757	3169–4328	−0.03270	3035	2598–3474	1.238	1.200–1.275
1.0	0.7	1.9	1.9	2.0	99.26	−0.02642	3757	3169–4328	−0.03280	3026	2590–3461	1.242	1.203–1.280
1.0	0.7	1.9	2.0	1.9	99.26	−0.02642	3757	3169–4328	−0.03280	3027	2591–3463	1.241	1.203–1.280
1.0	0.7	1.9	2.0	2.0	99.26	−0.02642	3757	3169–4328	−0.03290	3017	2583–3451	1.245	1.206–1.284
1.0	0.7	2.0	1.9	1.9	99.26	−0.02642	3757	3169–4328	−0.03300	3008	2576–3440	1.249	1.210–1.287
1.0	0.7	2.0	1.9	2.0	99.26	−0.02642	3757	3169–4328	−0.03310	2999	2569–3429	1.253	1.213–1.292
1.0	0.7	2.0	2.0	1.9	99.26	−0.02642	3757	3169–4328	−0.03309	3000	2569–3430	1.253	1.212–1.292
1.0	0.7	2.0	2.0	2.0	99.26	−0.02642	3757	3169–4328	−0.03319	2990	2562–3419	1.256	1.215–1.297

Note: All estimates are based on a 1.5% discounting rate. $\alpha_{1}$—Odds ratio for overweight. $\alpha_{2}$—Odds ratio for obesity. $\psi_{1}$—Odds ratio for meeting the recommended physical activity level. $\lambda_{1}$—Odds ratio for meeting the recommended fruit consumption. $\tau_{1}$—Odds ratio for meeting the recommended vegetable consumption.

**Table 4:** Estimated return on investment, considering effects on all four risk factors compared to weight status alone.

Program Effect on						Considering only Weight Status			Considering All 4 Risk Factors			Ratio (ROI_1_/ROI_2_)	
Overweight (α_1_)	Obesity (α_2_)	Physical Activity (ψ_1_)	Fruit (λ_1_)	Vegetables (τ_1_)	Cost	Cost Savings †	ROI_1_ (%)	95% CI	Cost Savings †	ROI_2_ (%)	95% CI	Ratio	95% CI
1.0	0.7	1.0	1.0	1.0	99.26	321.615	324%	281–384%	321.615	324%	281–384%	1.000	1.000–1.000
1.0	0.7	1.0	1.0	1.1	99.26	321.615	324%	281–384%	323.273	326%	283–386%	0.995	0.993–0.996
1.0	0.7	1.0	1.1	1.0	99.26	321.615	324%	281–384%	323.668	326%	283–386%	0.994	0.991–0.995
1.0	0.7	1.0	1.1	1.1	99.26	321.615	324%	281–384%	325.326	328%	285–388%	0.989	0.985–0.991
1.0	0.7	1.1	1.0	1.0	99.26	321.615	324%	281–384%	329.250	332%	288–393%	0.977	0.974–0.980
1.0	0.7	1.1	1.0	1.1	99.26	321.615	324%	281–384%	330.911	333%	290–394%	0.972	0.968–0.976
1.0	0.7	1.1	1.1	1.0	99.26	321.615	324%	281–384%	331.304	334%	290–395%	0.971	0.967–0.975
1.0	0.7	1.1	1.1	1.1	99.26	321.615	324%	281–384%	332.965	335%	292–396%	0.966	0.961–0.971
1.0	0.7	1.9	1.9	1.9	99.26	321.615	324%	281–384%	398.108	401%	350–469%	0.808	0.784–0.833
1.0	0.7	1.9	1.9	2.0	99.26	321.615	324%	281–384%	399.362	402%	352–470%	0.805	0.781–0.831
1.0	0.7	1.9	2.0	1.9	99.26	321.615	324%	281–384%	399.254	402%	352–470%	0.806	0.781–0.832
1.0	0.7	1.9	2.0	2.0	99.26	321.615	324%	281–384%	400.510	403%	353–471%	0.803	0.779–0.829
1.0	0.7	2.0	1.9	1.9	99.26	321.615	324%	281–384%	401.698	405%	354–473%	0.801	0.777–0.827
1.0	0.7	2.0	1.9	2.0	99.26	321.615	324%	281–384%	402.954	406%	355–474%	0.798	0.774–0.825
1.0	0.7	2.0	2.0	1.9	99.26	321.615	324%	281–384%	402.845	406%	355–474%	0.798	0.774–0.825
1.0	0.7	2.0	2.0	2.0	99.26	321.615	324%	281–384%	404.101	407%	356–475%	0.796	0.771–0.823

Note: All estimates are based on a 1.5% discounting rate. †—Based on $12,174 as annual health care costs attributable to chronic disease (Table S6). $\alpha_{1}$—Odds ratio for overweight. $\alpha_{2}$—Odds ratio for obesity. $\psi_{1}$—Odds ratio for meeting the recommended physical activity level. $\lambda_{1}$—Odds ratio for meeting the recommended fruit consumption. $\tau_{1}$—Odds ratio for meeting the recommended vegetable consumption. ROI—Return on investment.

## Supplementary Materials

The following are available online at https://www.mdpi.com/2072-6643/12/4/1101/s1, Table S1: Joint distribution of weight status, physical activity, fruit and vegetable consumption among under 14-year old’s in Canada, Table S2: Multivariate model for the joint transition probabilities of weight status, physical activity, fruit and vegetable consumption, Table S3: Effect of chronic diseases on all-cause mortality, Table S4: Effect of weight status, physical activity, fruit and vegetable consumption on all-cause mortality, Table S5: Impact of chronic diseases and weight status on health-related quality of life, Table S6: Estimated attributable annual direct costs per person with chronic disease, Table S7: Reference values use for meeting physical activity, fruit and vegetable consumption.
