# Supplementary material for: An Enhanced Approach for Economic Evaluation of Long-Term Benefits of School-Based Health Promotion Programs
Source: Nutrients. 2020 Apr 16;12(4):1101. doi: 10.3390/nu12041101 (PMC7230436; doi:10.3390/nu12041101)
Supplement: Supplementary file 1 [file nutrients-12-01101-s001.pdf]

Supplementary Tables.

Table S1. Joint distribution of weight status, physical activity, fruit and vegetables consumption among under 14-year old's in Canada.

Weight Status	Physical activity	Fruit consumption	Vegetables consumption	Proportion	
				Male	Female
Normal	-	-	-	0.17249	0.24409
Normal	-	-	+	0.01873	0.02492
Normal	-	+	-	0.06900	0.11821
Normal	-	+	+	0.02001	0.03633
Normal	+	-	-	0.25844	0.17723
Normal	+	-	+	0.02968	0.02348
Normal	+	+	-	0.14741	0.16509
Normal	+	+	+	0.05090	0.05766
Overweight	-	-	-	0.05178	0.03886
Overweight	-	-	+	0.00380	0.00324
Overweight	-	+	-	0.01615	0.02212
Overweight	-	+	+	0.00510	0.00494
Overweight	+	-	-	0.04837	0.02336
Overweight	+	-	+	0.00580	0.00255
Overweight	+	+	-	0.03456	0.01828
Overweight	+	+	+	0.01257	0.00706
Obese	-	-	-	0.01880	0.00910
Obese	-	-	+	0.00151	0.00072
Obese	-	+	-	0.00565	0.00608
Obese	-	+	+	0.00057	0.00094
Obese	+	-	-	0.01780	0.00756
Obese	+	-	+	0.00146	0.00041
Obese	+	+	-	0.00726	0.00501
Obese	+	+	+	0.00213	0.00274

Note: + Meeting recommendations, - not meeting recommendations

Table S2. Multivariate model for the joint transition probabilities of weight status, physical activity, fruit and vegetables consumption

Outcome		*Explanatory variable*	*Coefficient*	*Estimate*	*S.E*
Fruit consumption	F+	Intercept	β_1	-2.3611613	0.0058680
		Fruit consumption	β_2	2.1163706	0.0016393
		Vegetables consumption	β_3	0.6451192	0.0046017
		Female	β_4	0.6178274	0.0011011
		Age	β_5	-0.0560573	0.0004345
		Age^2	β_6	0.0014815	0.0000097
		Age^3	β_7	-0.0000105	0.0000001
		Fruit consumption × Female	β_8	-0.2047442	0.0020195
		Vegetables consumption × Female	β_9	-0.3729756	0.0050889
Physical activity	P+	Intercept	β_{10}	-0.9055989	0.0012925
		Vegetables consumption	β_{11}	0.1465257	0.0019465
		Female	β_{13}	-0.2505377	0.0007775
		Age	β_{14}	-0.0103348	0.0000257
		Physical activity	β_{12}	1.5149704	0.0017933
		Age × Physical activity	β_{15}	0.0066474	0.0000389
		Obese × Female	β_{16}	-0.1570425	0.0019136
		Obese	β_{17}	0.1374311	0.0016494
Vegetables consumption	V+	Intercept	β_{18}	-5.2173980	0.0105138
		Fruit consumption	β_{19}	-0.3932852	0.0051870
		Vegetables consumption	β_{20}	2.3697098	0.0053781
		Female	β_{22}	1.5505861	0.0117453
		Age	β_{23}	0.0000405	0.0004588
		Age^2	β_{25}	0.0000964	0.0000048
		Vegetables consumption × Female	β_{29}	-0.2400739	0.0057869
		Physical activity	β_{21}	0.5488138	0.0036502
		Age × Female	β_{24}	0.0246083	0.0005254
		Age × Fruit consumption	β_{26}	0.0129976	0.0001046
		Age^2 × Female	β_{27}	-0.0005552	0.0000056
		Physical activity × Female	β_{28}	-0.4088366	0.0040422
Weight status	Obese	Intercept	β_{30}	-1.3892126	0.0113482
		Female	β_{32}	-0.5139036	0.0041988

		Age	β_{34}	-0.2238394	0.0008285
		Age^2	β_{42}	0.0050025	0.0000180
		Age^3	β_{44}	-0.0000362	0.0000001
		Obese	β_{40}	9.7328316	0.0047080
		Age × Female	β_{36}	0.0050243	0.0000853
		Overweight	β_{38}	4.8518591	0.0028125
	Overweight	Intercept	β_{31}	-1.3289219	0.0059517
		Female	β_{33}	-0.8428883	0.0024616
		Age	β_{35}	-0.0325501	0.0004501
		Age^2	β_{43}	0.0012516	0.0000101
		Age^3	β_{45}	-0.0000118	0.0000001
		Obese	β_{41}	4.7299026	0.0040072
		Age × Female	β_{37}	0.0056846	0.0000514
		Overweight	β_{39}	3.6757343	0.0010109
Fruit consumption × Physical activity	F+, P+	Intercept	β_{46}	0.4237683	0.0009298
Fruit consumption × Vegetables consumption	F+, V+	Intercept	β_{47}	1.0879607	0.0022794
Physical activity × Vegetables consumption	P+, V+	Intercept	β_{48}	0.4667657	0.0021605
Physical activity × Weight status	P+, Obese	Intercept	β_{49}	-0.4763585	0.0014432
	P+, Overweight	Intercept	β_{50}	-0.2012455	0.0007636
Fruit consumption × Physical activity × Vegetables consumption	F+, P+, V+	Intercept	β_{51}	-0.1882117	0.0032406

Note: P+ meeting physical activity recommendations
F+ - Meeting fruit consumption recommendations
V+ - Meeting vegetables consumption recommendations

Table S3. Effect of chronic diseases on all-cause mortality.

Source	Chronic disease	Age group	Male	Female
Preis et al, 2009[26]	Diabetes	45-74	1.81	2.29
Robitaille et al, 2012[27]	Hypertensive heart disease	20-24	4.2	4.2
		25-29	4	4
		30-34	2.7	2.7
		35-39	2.4	2.4
		40-44	2.4	2.4
		45-49	1.9	1.9
		50-54	1.8	1.8
		55-59	1.6	1.6
		60-64	1.5	1.5
		65-69	1.5	1.5
		70-74	1.4	1.4
		75-79	1.2	1.2
		80-84	1.2	1.2
		85+	1.1	1.1
Vandentorren et al,2003[28]	Asthma	25-59	1.15	1.22
Bronnum-Hansen et al, 2001[29]	Ischemic heart disease	25+	2.58	2.85
	Ischemic stroke	25+	2.58	2.85
	Hemorrhagic stroke	25+	2.58	2.85
Gibertoni et al, 2015[30]	Chronic kidney disease	1+	1.41	1.41
Canadian Cancer Society, 2015[31]	Leukemia	15-99	1.67	1.69
	Breast cancer	15-99	1.25	1.14
	Colon and rectum cancer	15-99	1.56	1.54
	Esophageal cancer	15-99	7.69	6.67
	Kidney cancer	15-99	1.49	1.45
	Larynx cancer	15-99	1.59	1.56
	Lip and oral cavity cancer	15-99	1.64	1.47
	Liver cancer	15-99	5	5.26
	Multiple myeloma	15-99	2.27	2.44
	Non-Hodgkin's lymphoma	15-99	1.54	1.47
	Ovarian cancer	15-99	1	2.22
	Pancreatic cancer	15-99	12.5	12.5
	Thyroid cancer	15-99	1.05	1.01
	Tracheal bronchus and lung cancer	15-99	7.14	5
	Uterine cancer	15-99	1	1.18
Nuesch et al, 2011[32]	Osteoarthritis	35+	1.58	1.52
Choi et al, 2007[33]	Gout	1+	1.28	1.28
Fernandez et al, 2017[34]	Low back pain	70+	1.13	1.13
Khanna, 2013[35]	Cataract	1+	1.58	1.58
Ruhl et al, 2011[36]	Gallbladder and biliary diseases	20+	1.3	1.3
Andersson, 2013[37]	Atrial fibrillation and flutter	<65	2.15	1.76
		65-74	1.72	1.36
		75-85	1.44	1.24
Ganguli et al, 2005[38]	Alzheimer's disease and other dementias	65+	1.4	1.4
Statistics Canada, 2012[39]	Gallbladder and biliary tract cancer	15+	5	5

	Nasopharynx cancer	15+	1.75	1.47
	Other pharynx cancer	15+	1.69	1.75

Table S4. Effect of weight status, physical activity, fruit and vegetables consumption on all-cause mortality

**Source**	**Risk Factor**	**Age group**	**Male**		**Female**	
			RR	95%CI	RR	95%CI
Flegal et al., 2013 [59]	**Weight Status**					
Meta-Analysis	Overweight	All	0.94	0.91-0.96	0.94	0.91-0.96
	Obesity	All	1.18	1.12-1.25	1.18	1.12-1.25
Guenther et al, 2011[60]	Sufficient total physical activity (7-MET-h/day)	All	0.83	0.78-0.89	0.79	0.74-0.85
Computed from results published by Aune et al, 2017[61]	Adequate fruit consumption	<15	0.92	0.89-0.95	0.94	0.91-0.96
		15-54	0.87	0.83-0.92	0.88	0.84-0.93
		55+	0.88	0.84-0.93	0.88	0.84-0.93
	Adequate vegetables consumptions	<15	0.93	0.90-0.96	0.93	0.90-0.96
		15-54	0.80	0.73-0.88	0.85	0.79-0.90
		55+	0.85	0.79-0.90	0.85	0.79-0.90

Table S5. Impact of chronic diseases and weight status on health related quality of life.

Source	disease	Impact	95%CI
Schultz et al, 2003[43]	Diabetes	-0.06	-0.07 , -0.04
	Hypertensive heart disease	-0.01	-0.02 , 0.00
	Asthma	-0.02	-0.03 , -0.01
	Ischemic heart disease	-0.06	-0.08 , -0.05
	Ischemic stroke	-0.17	-0.22 , -0.13
	Hemorrhagic stroke	-0.17	-0.22 , -0.13
	Chronic kidney disease	-0.09	-0.10 , -0.07
	Leukemia	-0.02	-0.04 , 0.00
	Osteoarthritis	-0.09	-0.10 , -0.07
	Gout	-0.09	-0.10 , -0.07
	Low back pain	-0.06	-0.07 , -0.06
	Cataract	-0.08	-0.11 , -0.06
	Gallbladder and biliary diseases	-0.09	-0.10 , -0.07
	Atrial fibrillation and flutter	-0.09	-0.10 , -0.07
	Alzheimer's disease and other dementias	-0.34	-0.42 , -0.26
	Breast cancer	-0.02	-0.04 , 0.00
	Colon and rectum cancer	-0.02	-0.04 , 0.00
	Esophageal cancer	-0.02	-0.04 , 0.00
	Gallbladder and biliary tract cancer	-0.02	-0.04 , 0.00
	Kidney cancer	-0.02	-0.04 , 0.00
	Larynx cancer	-0.02	-0.04 , 0.00
	Lip and oral cavity cancer	-0.02	-0.04 , 0.00
	Liver cancer	-0.02	-0.04 , 0.00
	Multiple myeloma	-0.02	-0.04 , 0.00
	Nasopharynx cancer	-0.02	-0.04 , 0.00
	Other pharynx cancer	-0.02	-0.04 , 0.00
	Non-Hodgkin's lymphoma	-0.02	-0.04 , 0.00
	Ovarian cancer	-0.02	-0.04 , 0.00
	Pancreatic cancer	-0.02	-0.04 , 0.00
	Thyroid cancer	-0.02	-0.04 , 0.00
	Tracheal bronchus and lung cancer	-0.02	-0.04 , 0.00
	Uterine cancer	-0.02	-0.04 , 0.00
Jia et al., 2005[44]	Over weight	-0.013	
	Class I Obesity	*-0.033*	
	Class II Obesity	*-0.073*	
	Obesity(Weighted average)	-0.047	

Table S6. Estimated attributable annual direct costs per person with chronic disease

Chronic disease diagnosis category	Prevalence in 2016(%)	Estimated number of cases in 2016	Attributed direct costs in 2008 (Million $)†	% of Total 2008 costs	Estimated direct costs in 2016 (Million $)†	Annual costs per case
Malignant Neoplasms	1.8	645,221	3,994.7	4.7	7,854.6	12,174
Cardiovascular Diseases	10.0	3,636,602	11,785.4	14.0	23,173.3	6,372
Diabetes Mellitus	30.6	11,097,412	2,190.5	2.6	4,307.1	388
Digestive Diseases	4.8	1,740,324	5,530.6	6.6	10,874.6	6,249
Musculoskeletal Diseases	27.2	9,857,261	5,783.3	6.9	11,371.6	1,154
Neuropsychiatric Conditions	36.3	13,166,571	11,437.7	13.6	22,489.7	1,708
Respiratory Diseases	10.9	3,963,458	3,659.7	4.3	7,196.0	1,816
Sense Organ Diseases	71.0	25,747,087	2,132.9	2.5	4,193.9	163
**Total direct costs in respective years.** Including diagnosis categories not listed here			**84,349.6**		**165,854.5**	

Note: †Includes only costs for drugs, physician, hospital and other Institutions.

Table S7. Reference values use for meeting physical activity, fruit and vegetables consumption.

Factor	Age	Male	Female
Fruit (servings/day)	≤14	2	2
	15-54	3	3
	55+	3	3
Vegetable (servings/day)	≤14	3	3
	15-54	5	4
	55+	4	4
Physical activity(minutes of moderate-vigorous activity per week)	12-17	420	420
	18-64	150	150
	65+	150	150
